# Chitinase: diversity, limitations, and trends in engineering for suitable applications

**DOI:** 10.1042/BSR20180323

**Published:** 2018-08-29

**Authors:** Ayokunmi Oyeleye, Yahaya M. Normi

**Affiliations:** 1 Department of Cell and Molecular Biology, Faculty of Biotechnology and Biomolecular Sciences, Universiti Putra Malaysia, 43400 Serdang, Malaysia; 2 Enzyme and Microbial Technology Research Center, Faculty of Biotechnology and Biomolecular Sciences, Universiti Putra Malaysia, 43400 Serdang, Malaysia

**Keywords:** biocontrol, chitooligosaccharides, Chitinases, protein engineering, variability

## Abstract

Chitinases catalyze the degradation of chitin, a ubiquitous polymer generated from the cell walls of fungi, shells of crustaceans, and cuticles of insects. They are gaining increasing attention in medicine, agriculture, food and drug industries, and environmental management. Their roles in the degradation of chitin for the production of industrially useful products and in the control of fungal pathogens and insect pests render them attractive for such purposes. However, chitinases have diverse sources, characteristics, and mechanisms of action that seem to restrain optimization procedures and render standardization techniques for enhanced practical applications complex. Hence, results of laboratory trials are not usually consistent with real-life applications. With the growing field of protein engineering, these complexities can be overcome by modifying or redesigning chitinases to enhance specific features required for specific applications. In this review, the variations in features and mechanisms of chitinases that limit their exploitation in biotechnological applications are compiled. Recent attempts to engineer chitinases for improved efficiency are also highlighted.

## Introduction

Enzymes drive metabolic reactions that would otherwise remain in prolonged states of inactivity, with specificity, speed and efficiency. They have been studied for ages and there is an increasing knowledge about their enormous capabilities that is opening up the world of research in enzyme engineering for enhanced activities as well as for the development of new enzymatic functions.

Chitinases are among enzymes of useful practical applications because of the key roles they play in the degradation of crystalline polysaccharides. They possess interesting features that render them attractive for protein engineering studies and are gaining increasing attention among researchers. Chitinases catalyze the degradation of β-1→4-linkages in chitin (C_8_H_13_O_5_N)*_n_*, an abundant polymer second only to cellulose and a major component of the structural framework of fungal cell walls [1], the exoskeleton and gut lining of insects, and the shells of crustaceans [2,3]. They are a diverse group of enzymes with varying structures and mechanisms that determine their activity and suitability for field applications such as in the: control of plant diseases and insect pests [4]; synthesis of chitooligosaccharides (COS) [5] for use in the food and drug industries; management of marine wastes; and biofuel production [6,7].

Although the classification, characteristics and applications of chitinases have been extensively reviewed by a number of authors [4,6,8–12], none closely relates the diversity of chitinases as a possible limiting factor to their application. This review therefore highlights and emphasizes the diversity in sources, structural features, and mechanistic properties as major limitations to the development and utilization of chitinases in industrial and field applications. In addition, attempts that have been made toward fine-tuning structural features and enhancing catalytic mechanisms of chitinases through engineering techniques are compiled.

## Classification of chitinases based on amino acid sequences and domain architectures

**Figure 1:**
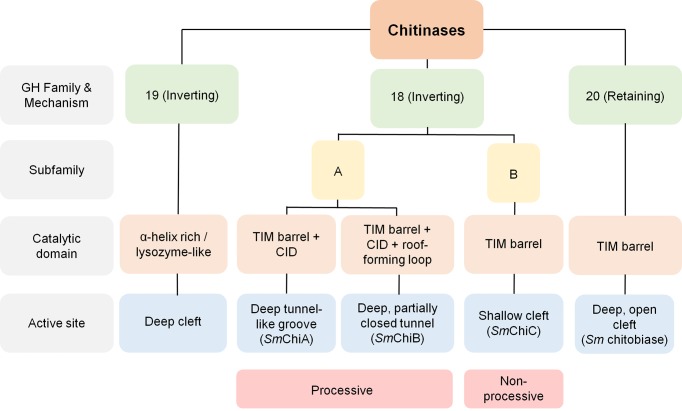
Classification of bacteria chitinases based on mechanism of activity and shape of catalytic domain

Chitinolytic enzymes are classified in the CAZY database [13] into glycosyl hydrolase (GH) families 18, 19, and 20 as shown in Figure 1. They differ markedly in their amino acid sequences and catalytic properties [14], and although there is an inherent ambiguity in this taxonomy [15], these three families have been biochemically validated to consist of enzymes having chitinolytic properties. Families 18 and 19 glycosidases that are subjects of this review are regarded as chitinases, because they catalyze the degradation of chitin polymers. GH family 20 includes chitobiase and β-N-acetylhexosaminidase that catalyze the breakdown of dimeric units of N-acetylglucosamine (Chitobiose), terminal N-acetylgalactosamine, or glucosamine from glyco-conjugates [4,16]. All family 18 chitinases are commonly characterized by a catalytic region that consists of a triosephosphate isomerase (TIM) barrel (β/α)_8_ domain [17–20], while the catalytic domain of family 19 is an α-helix rich lysozyme-like domain characterized by a deep cleft [21].

Although the structure of the catalytic sites broadly describes each family, other distinct variations are found in the topologies of members of both families as highlighted in the following sections.

### Diversity of GH family 18 chitinases

The GH family 18 chitinases have been characterized from diverse chitin and non-chitin producers. Bacteria, fungi, and insect chitinases are the most diverse members of this family. These members have evolved a highly efficient mechanism of chitin hydrolysis in which a cocktail of chitinases are secreted synergistically to degrade chitin for developmental functions or as defense mechanisms [11]. This synergistic property has been proven experimentally especially in *Serratia marcescens* [22], which produces up to four chitinolytic enzymes. Bacterial chitinases are divided into subfamilies A and B. These groups differ only in the presence of a chitin insertion domain (CID) found in subfamily A. Bacteria producing GH family 18 chitinases include the genera *Serratia* [23,24], *Vibrio* [25], *Bacillus* [26], *Chromobacterium* [27], and *Yersinia* [28]*.*

Like bacterial chitinases, fungal chitinases are diverse and until recently, all were known to exclusively belong to the GH family 18 [1,29]. Despite vast research on fungal chitinases, their taxonomy relies strongly on plant and bacterial chitinases. Hence, three subgroups A, B, and C were proposed (reviewed in [9]). Chitinases have been well characterized from *Trichoderma artroviridae* [30], *Aspergillus nidulans* [30], and *Aspergillus fumigatus* [31].

GH family 18 chitnases are also predominant among plants. Generally, all plant chitinases are grouped into classes I–V with only classes III and V having sequence and structural homology with the GH family 18 chitinases. Examples of class III are chitinases from *Hevea brasiliensis* [32] and the fern *Pteris ryukyuensis* (*Pr*ChiA) [33], while class V includes chitinases from *Arabidopsis thaliana* (*At*ChiA) [34] and *Nicotiana tabacum* (*Nt*ChiA) [35].

### Diversity of GH family 19 chitinases

GH family 19 chitinases have been identified mainly from plants, several bacteria, very few viruses (CAZy database, http://www.cazy.org) and only one fungi [29]. Members of this family are thought to be produced as part of a defense mechanism against fungal pathogens. Classes I, II, and IV of plant chitinases belong to GH family 19 including the well-characterized chitinases from *Oryza sativa* [36], *Bryum coronatum* [37] and *Picea abies* [38] respectively. These classes differ in the presence (class I) or absence (class II) of a chitin-binding domain (CBD). Class IV are smaller because of the absence of some extra loops in their structure [32,39].

Reports on the structural and mechanistic properties of this family are largely available for the plant members, but not much has been done with respect to those of the bacterial members apart from the well-studied chitinase from *Streptomyces griseus* (*Sg*ChiC). *Streptomyces griseus* and *Streptomyces coelicolor* are the only two representatives of the bacterial family 19 chitinases with solved 3D structures. Table 1 is a list of some chitinases with 3D structures. *Sg*ChiC was previously the only known non-plant chitinase of the GH family 19 described, characterized decades after the classification of plant chitinases. Others were identified much later from other actinobacterial and non-bacterial species including protozoans and metazoans [40]. Very recently, the first fungal GH family 19 chitinase was isolated and characterized from *Nosema bombycis* (designated as *Nb*ChiA). It is currently the only known fungal GH family 19 chitinase [29] identified more than two decades after the characterization of the first bacterial GH family 19 chitinase. The wide gap in time during which new family 19 chitinases were discovered suggests the possible existence of a greater diversity of sources and the ability of more organisms to evolve the ability to produce new members of this family.

**Table 1:** Some examples of chitinases with 3D structures in the Protein Data Bank

Source	Gene name*	PDB ID	Mechanism	Activity*	Active site topology	Reference
*Serratia marcescens*	ChiB	1E15	retaining	exo-	partially closed tunnel	[27]
*Streptomyces griseus* HUT6037	ChiC	1WVU	inverting	endo-	deep cleft	[34]
*Streptomyces coelicolor*	ChiG	2CJL	inverting	endo-	deep cleft	[61]
*Brassica juncea*	Chi3	2Z37	inverting	endo-	deep, wide cleft	[62]
*Carica papaya*	ChiC	3CQL	inverting	endo-	deep cleft	[63]
*Picea abies*	Chia4-Pa2	3HBD	inverting	endo-	deep cleft	[64]
*Clonastachys rosea*	Chi1	3G6L	retaining	endo-	tunnel shape	[65]
*Oryza sativa* L japonica	ChiA1b	3IWR	inverting	endo-	deep, long cleft	[66]
*Bacillus cereus* NCTUR	ChiNCTU2	3N11	retaining	endo	deep cleft	[67]
*Nicotiana tabacum*	ChiV	3ALF	retaining	endo-	tunnel shape	[68]
*Arabidopsis thaliana*	ChiC	3AQU	retaining	exo-/endo-	large open cleft	[69]
*Yersinia Entomophaga*	Chi1	3OA5	retaining	endo-	tunnel shape	[70]
*Crocus vernus*	NS	3SIM	retaining	NS	NS	Unpublished
*Aspergillus fumigatus*	ChiC	2Y8V	retaining	NS	NS	NC
*Serratia marcescens*	ChiC	4AXN	retaining	endo-	shallow cleft	[9]

* Stated in the Protein Data Bank in Europe (PDBe); NC, no citation available; NS, not stated.

In an extensive evolutionary study by [41], up to 20 unique conserved motifs were identified among GH family 19 chitinases, indicating the degree of diversification that has occurred over time. The study also revealed the evolutionary path of all GH family 19 chitinases as originating from plants. This includes chitinases from actinobacteria and purple bacteria as directly or indirectly acquired through a horizontal gene transfer. Eventually, a single motif common to all GH family 19 chitinases, ([FHY]-G-R-G-[AP]-x-Q-[IL]-[ST]-[FHYW]-[HN]-[FY]-NY), was revealed.

### Multiple chitinase production

Multiple chitinase production by a single species extends the diversity of chitinases as seen in several organisms. For instance, *S. marcescens* produces at least three different GH family 18 chitinases: ChiA, ChiB, and ChiC [23]. *Streptomyces coelicolor* has up to 13 types distributed between the GH families 18 and 19 chitinases [42]. *Paenibacillus* sp. Str. FPU-7 secretes at least seven chitinases [5], and some fungi are known to secrete ~20. The production of multiple chitinases in a single organism is presumed to be a result of gene acquisition through lateral transfer from one organism to the other [43]. The different chitinases may be secreted in such organisms to fulfill antagonistic, morphological, or nutritional roles or to function synergistically for the efficient hydrolysis of chitin as source of carbon and nitrogen.

## Diversity of roles in nature

Chitinases are expressed in various organisms including those that lack chitin such as plants, bacteria, viruses and vertebrates, as well as in chitin-containing organisms such as fungi, insects, and crustaceans. These organisms produce them for various physiological functions.

Bacteria are significantly involved in the degradation of chitin in nature, largely contributing to biogeocemical recycling and maintaining the balance between carbon and nitrogen in the ecosystem [44,45]. Chitinase production in bacteria can be indicative of virulence especially in entomopathogenic bacteria species [46]. They make use of chitin as sole carbon and energy source [47] by secreting a cocktail of chitinases during pathogenesis in chitin-containing hosts or during the hydrolysis of chitinous waste.

Like bacteria, fungi make use of an efficient chitinolytic system. They produce chitinases at different phases during their growth. Fungal chitinases play key roles in nutrition, morphogenesis, autolysis, competition, defense, or parasitism. Mycoparasitic, nematophagous [48], and entomopathogenic fungi [9,31,49] have been reported to produce chitinolytic enzymes that contribute to their biocontrol potentials. The functions and biocontrol potential of chitinases are widely known and well-reviewed [50–52].

Insects are reported to possess a greater number of chitinases than any other organism [53]. Their chitinases are produced during ecdysis for the shedding of old cuticle. Recently, studies on the catalysis and structural characterization of Lepidoptera chitinase, *Of*ChtI from *Ostrinia furnacalis* demonstrated that the enzyme was indispensable to moulting [53,54].

Unlike others, plant chitinases seem to play more diverse and multiple physiological roles [35] and their production is induced in response to biotic and abiotic stress factors. Plant chitinases are tissue specific and can be developmentally regulated or produced in response to pathogenic invasion [37]. For example, *Nicotiana tabaccum* chitinase (*NtChiV*) gene expression is induced by viral infection with tobacco mosaic virus, wounding or ultraviolet irradiation, indicating that the enzyme can be induced by both abiotic and biotic factors. Similarly, *A. thaliana* chitinase (*AtChiC*) gene expression was reportedly induced by fungal pathogens, abscisic and jasmonic acids, flagellin, salt and osmosis, all of which are stress-associated factors in plants [17,35].

These diverse roles are a result of the different features among chitinases that may influence their universal or specific applications in industry, medicine, environment, or agriculture.

## Structural differences among chitinases

### Active site topologies

**Figure 2:**
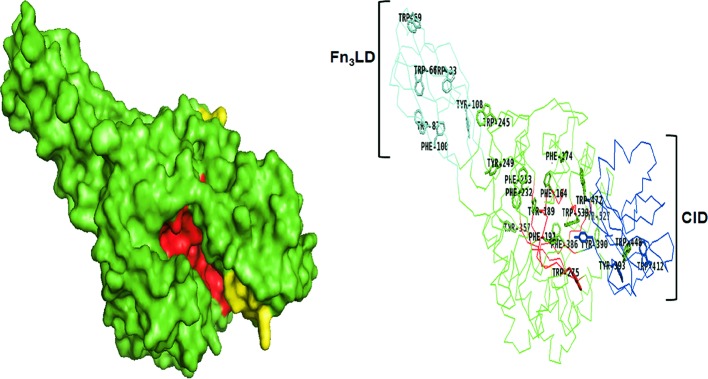
*Sm*ChiA (PDB ID:1EDQ). Surface model shows deep, open tunnel-like groove (red) with a short loop (yellow). Ribbon model shows the Fn_3_LD (cyan) and CID (blue) extending the substrate-binding site which is lined with aromatic residues (shown as sticks) from the N-terminus to the C-terminus.

**Figure 3:**
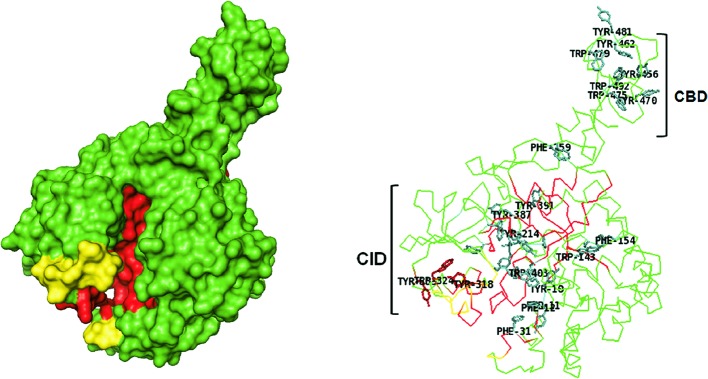
*Sm*ChiB (PDB ID: 1E15) Surface model shows deep, tunnel-like catalytic groove (red), with loops (yellow ) forming a partially closed roof. Ribbon model shows extended substrate-binding groove rich in aromatic residues (shown as sticks), lined from the N-terminus to the C-terminus.

**Figure 4:**
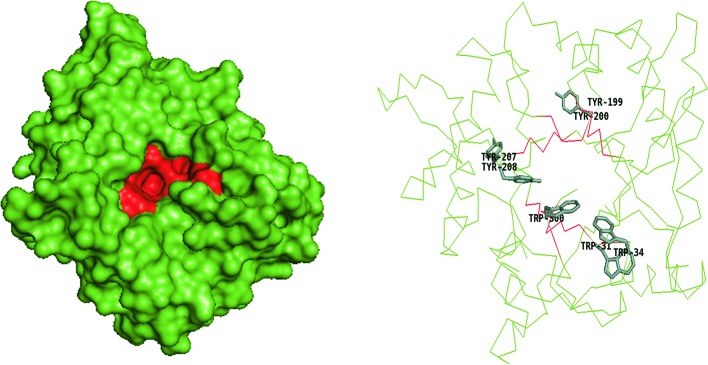
Catalytic domain of non-processive *Sm*ChiC (PDB ID: 4AXN) Surface model with pocket shaped cleft (red) and ribbon model showing few aromatic residues (green sticks).

**Figure 5:**
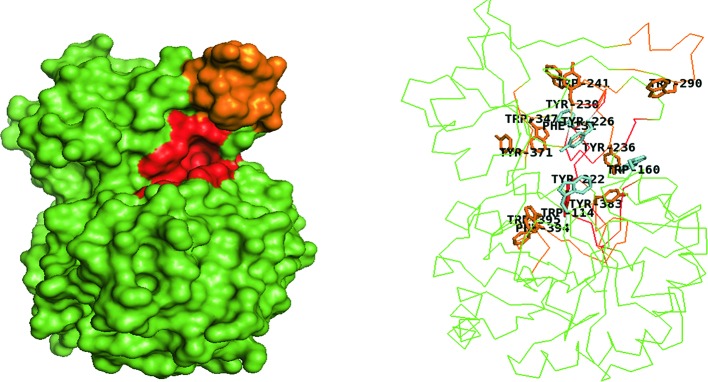
Catalytic domain of a transglycosylating chitinase, *Sp*ChiD PDB ID: 4NZC Surface model shows an open, shallow cleft (red), restricted by a loop (orange). Ribbon model shows more aromatic residues (orange sticks) buried within the loops obstructing the catalytic cleft than surface exposed aromatic residues (green sticks).

**Figure 6:**
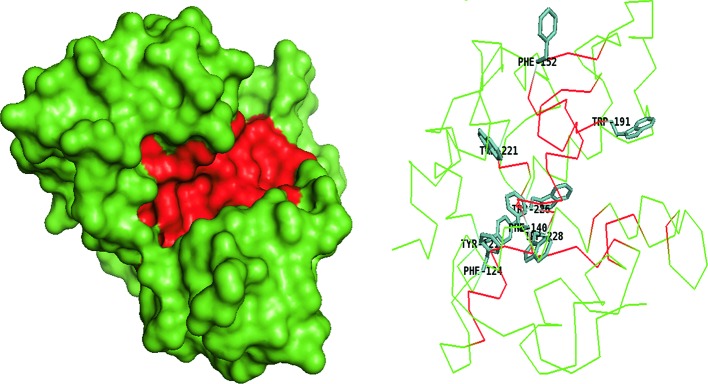
Catalytic domain of *Sg*ChiC (PDB ID: 1WVU) Surface model shows wide and deep cleft (red) found in GH family 19 chitinases. Ribbon model reveals few aromatic residues (green sticks) in the deep cleft.

Variations in topology of catalytic clefts among chitinases are represented in surface and ribbon models as depicted in Figures 2–6. Generally, the active site of GH could be tunnel (groove-like) (Figures 2 and 3), deep, or pocket shaped clefts (Figures 4–6). Tunnel and pocket shaped clefts are common topologies found in GH 18 chitinases. These define substrate specificities as well as lengths of the substrate and cleavage subsites in chitinases [55,56]. The tunnel shape is thought to have evolved from the open cleft topology by the formation of long loops that form a partial covering called ‘the roof’ over the cleft [57]. This can be observed by the regions colored yellow in *Sm*ChiA (Figure 2) and *Sm*ChiB (Figure 3), which depicts a difference in position and length of loops that extend longer in *Sm*ChiB, forming a roof covering the tunnel. The extra loops enable the active site to undergo conformational changes that trigger an ‘open’ and ‘close’ conformation of the catalytic groove [57,58].

Some family 18 chitinases consist of an extra domain within the catalytic domain called the CID, which defines the depth and topology of the active site. The CID is an (α+β) domain found only in subfamily A of family 18 chitinases, sandwiched between the seventh and eighth β-strands of the TIM barrel fold of the catalytic site [59]. The extensively studied chitinases from *S. marcescens* designated as *Sm*ChiA, *Sm*ChiB, and *Sm*ChiC have been applied as models for describing other chitinases especially those of the family 18 glycosidases (Figure 1). *Sm*ChiA and *Sm*ChiB both belong to the subfamily A. Their TIM barrel catalytic domains house the CID that has been postulated to be responsible for the tunnel-like deep clefts and processivity in chitin degradation (Figures 1–3) [44]. The CID is absent in *Sm*ChiC suggesting that its catalytic cleft is more open and shallow compared to its counterparts (Figure 4) [74].

GH 19 chitinases generally have a wide and open cleft predominantly composed of loops and α-helices (Figure 6). The number of loops and helices found in their catalytic domains varies among members and are thus described as ‘loopful’ or ‘loopless’ [17,60]. In the loopful chitinases, extra loops are located on both sides of the substrate-binding cleft, while the loopless members lack these extra loops [17,61]. The extra loops are thought to extend the substrate-binding grooves providing more subsites for binding to longer chitin chains [38,60].

### Auxilliary chitin-binding domains

**Figure 7:**
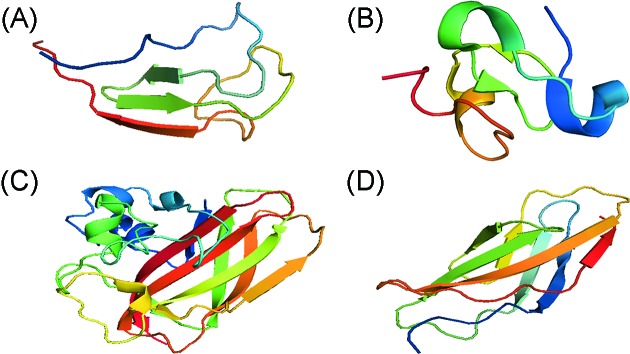
Putative CBDs among chitinases (**A**) CBD (belonging to .CBM-5) of *Streptomyces griseus* chitinase C (PDB ID: 2D49). (**B**) Chitin-binding domian (belonging to CBM-18) of a chitinase-like protein from *H. brasiliensis* (PDB ID: 4MPI). (**C**) Chitin-binding protein (CBP-21) of *S. marcescens* (PDB ID: 2LHS). (**D**) Fn_3_LD of *Bacillus circulans* WL-12 chitinase A1 (PDB ID: 1K85).

Chitinases may consist of other domains (Figure 7) some of which are assigned a chitin-binding role because they can adhere to and disrupt the packing of chitin crystals [62–64]. Although the extent to which the CBD influences the activity of chitinases is not clearly known, these extra domains are found to adopt varying locations and structural properties that contribute to differences in hydrolytic properties [65]. Studies of chitinases without CBD and those with deleted CBD have shown that the auxiliary domain may contribute to partial hydrolysis of substrate [66] and correct positioning of substrates in active sites or processivity [16,25].

*Sm*ChiA and *Sm*ChiB are known to consist of a fibronectin III-like domain (Fn_3_LD) and CBD respectively (Figures 2 and 3), while *Sm*ChiC has both located at the C-terminus (Figure 4) [22]. Although both *Sm*ChiA and *Sm*ChiB have similar active site topologies, they exhibit varying specific activities and substrate specificities that are linked to the locations of their extra domains. *Sm*ChiA that has its auxiliary (Fn_3_LD) domain located on its N-terminus degrades chitin from the reducing end, while *Sm*ChiB with its C terminus CBD degrades chitin from the non-reducing end [68].

Brurberg et al. [69] proposed that the N-terminal Fn_3_LD provides an extended substrate-binding site that permits the entry of two or more trimeric substrate molecules. The CBD of *Sm*ChiB on the other hand is different in amino acid sequence and structure. It belongs to the family 5 of carbohydrate-binding modules (CBM) and also contains two conserved solvent exposed tryptophan amino acid residues believed to enhance substrate binding and hydrolysis [70,71].

Li et al. [72] demonstrated through site-directed mutagenesis (SDM) of Trp70, Trp245, and Ser33 in the CBD of *Aeromonas caviae* CB101 Chi1 that enzyme activity decreased toward crystalline chitin from 5.24 U/µmol to less than 2 U/µmol and colloidal chitin (CC) from 68.55 U/µmol to less than 45 U/µmol. Deletion of the whole CBD caused the enzyme to lose almost all its activity toward crystalline chitin, but interestingly retained most of its activity toward CC. This suggests that the CBD was necessary for complete degradation of insoluble crystalline chitin and not necessary for colloidal or soluble substrates.

GH family 19 also consists of multimodular members. Classes I and II of plant family 19 chitinases are highly similar except that class II lack CBDs [36]. *Streptomyces griseus* HUT6037 ChiC has an N-terminal CBD and a C-terminal catalytic domain connected by a linker peptide [21,34]. The N-terminal CBD of ChiC shares similarities with the family 5 cabohydrate-binding module (CBM5) found in some other GH family 18 bacteria chitinases [16]. Through deletion and SDM studies, the CBD of *S. griseus* ChiC was found to be important for chitin hydrolysis and antifungal activity [66,71].

Multiple CBDs and catalytic domains that may contribute to the diverse chitinase properties have also been reported. For example, the chitinase isolated from a metagenomic library designated as MetaChi18A has an N-terminal chitin Fn_3_LD followed by the GH-18 domain and two C-terminal CBDs joined to the catalytic domain by a polycystic kidney disease-like domain [73]. In addition, the recently solved 3D structure of ChiW from *Paenibacillus* sp. consists of six domains: a CBM-54, two GH family 18 catalytic sites with TIM barrel-like folds and CID’s, two immunoglobulin (Ig)-like fold domains with unknown functions and a glycine–serine rich linker connecting the CBM with the active sites [43].

## Classification based on differences in catalytic mechanisms

### Retaining versus inverting

**Figure 8:**
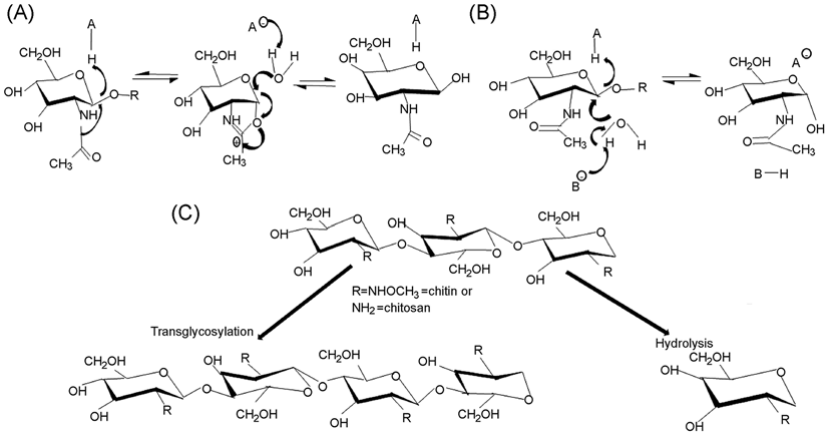
Mechanism of hydrolysis of glycosidic bonds in chitin (**A**) and (**B**) represent the chitinase enzymatic acid and nucleophile/base respectively. (A) Retention by a substrate-assisted mechanism. The glycosidic oxygen is first protonated by the catalytic acid (A), while nucleophilic assistance is provided by the N-acetyl oxygen. A glycosidic–enzyme intermediate becomes hydrolyzed by a water molecule leading to a second displacement that generates a product in which the configuration of the anomeric carbon in the substrate is retained (B). Inversion of the stoichiometry of the carbon anomer in the product. Protonation of the glycosidic oxygen by the acidic residue (A) takes place simultaneously with the activation of a water molecule by the catalytic base (B), thus yielding a product with a reversed stoichiometry different from the substrate. (**C**) Mechanism of TG and hydrolysis by chitinases. R represents NHOCH_3_ in chitin and NH_2_ in chitosan (deacetylated chitin).

Based on the stereochemical outcome of products of chitin hydrolysis, chitinases are categorized as either retaining or inverting. The GH family 18 chitinases are retaining and maintaining the configuration of the β-anomeric carbon of substrates in products (Figure 8A). This mechanism is attained by a substrate-assisted type of double displacement hydrolytic mechanism involving a DXXDXDXE [52,74] or simply DXDXE [17] (d-aspartic acid, e-glutamic acid, and X-any other amino acid) motif in the catalytic site. The general mechanism of GHs is known to involve two carboxylic acid residues, one serving as the general acid/base and the other as the nucleophile that stabilizes the oxazolinium intermediate in retaining enzymes or serves as a water-activating base in inverting enzymes [67]. In GH family 18, the substrate and not necessarily a second carboxylic acid residue plays the nucleophilic role, while the highly conserved glutamate residue positioned above the TIM barrel serves as the general acid/base, protonating the glycosidic oxygen and initiating the breakage of the glycosidic bond [14,16,75]. Jitonnom et al. [76] referred to the two stages involved in chitin hydrolysis by the retention mechanism as glycosylation and deglycosylation phases respectively. The glycosylation phase involves the transfer of a proton to the glycosidic oxygen (the leaving group) and the simultaneous nucleophilic assistance of the N-acetyl oxygen to the anomeric carbon leading to the cleavage of the glycosidic bond and the formation of the oxazolinium intermediate. The deglycosylation process, afterward initiated, involves the hydrolysis of the intermediate by a nucleophilic water molecule that is in close proximity to the anomeric carbon. This results in the overall retention of the configuration of the anomeric center [9,14].

The family 19 chitinases make use of the classical inverting mechanism (Figure 8B) common to several other glycosidases, in which there is a net inversion in the configuration of the anomeric carbon in the product [14]. Inverting chitinases hydrolyze glycosidic linkages by a one-step general acid/base hydrolytic mechanism putatively involving two glutamic acid residues on opposite sides of the deep catalytic cleft, one acting as the proton donor and the other as the nucleophile. The two glutamic acid residues involved in chitin hydrolysis are conserved in GH 19 chitinases. However, their distances vary among members. In bacteria family 19 chitinases, these residues are closer in the sequence because of the absence of a 13-residues loop found in the plant GH19 chitinases [77].

### Endo- versus exo-activity

Chitinases are described as been endo- or exo-acting based on the length of products formed during chitin degradation. Endo-chitinases cleave glycosidic bonds randomly along the chitin chain yielding dimers (diaceylchitobiose) and soluble low molecular mass multimers of N-acetyl-d-glucosamine (GlcNAc) such as chitotriose and chitotetraose. Exo-chitinases are either chitobiosidases that cleave chitin progressively from one end of the polymer releasing dimeric units or glucosaminidases that breakdown soluble oligomeric products of endo-chitinase degradation thereby generating monomers [41].

Exo- and endo-activities are two confusing features of chitinases that have steered the interest of researchers toward unraveling both mechanisms because the use of the length of products for classifying chitinases as endo- or exo- might be insufficient because enzymes previously thought to degrade chitin in an exo-fashion also possess some endo-activity as seen in *Sm*ChiA [22,56,69]. Although it is established that processive chitinases may degrade crystalline substrates in an exo-fashion, recent data reveal that the exo-active mode in processive chitinses is linked to better accessibility of chain ends [78] i.e. endo-mode for soluble substrates (chitosan) and exo-mode for crystalline substrates [79].

In addition, endo-chitinases are represented in both GH families 18 and 19. High-performance liquid chromatography (HPLC) analysis of the hydrolytic products generated from CC by *Sm*ChiA revealed the presence of GlcNAc, (GlcNAc)_2_ and (GlcNAc)_3_ while *Sm*ChiB produced (GlcNAc)_2_ and (GlcNAc)_3_ and *Sm*ChiC produced GlcNAc and (GlcNAc)_2_ in large amounts. The presence of GlcNAc along with (GlcNAc)_3_ as minor hydrolytic products was indicative of an endo-activity in *Sm*ChiA [22]. Sirkoski et al. [79], however, stated that all three chitinases were endo-acting when chitosan was used as a substrate and proposed that previous studies suggesting exo-active modes were in fact a result of the inaccessibility of the enzyme to the internal fibrils of chitin polymer. The study further suggested that loop structures in exo-active enzymes like ChiA and ChiB undergo distinct restructuring to enable endo-activity.

### Degree of processivity

Chitinases are further described as processive or non-processive based on their ability to catalyze multiple cycles of successive reactions while remaining attached to the substrate throughout the process [80]. Processive enzymes permit the threading of single-carbohydrate chains through the active-site cleft and the cleaving off of disaccharides at the catalytic center [57,79,80]. Non-processive enzymes on the other hand are detached from the chitin chain after every round of hydrolysis and are repositioned between successive rounds to enable productive binding. Processivity is a key characteristic of tunnel shaped chitinases involved in the hydrolysis of crystalline chitin. Their loops form a roof that enables an endo-hydrolysis or positioning of the enzyme close to a free chain end of the crystalline polymer [57] that makes the substrate to be threaded through the tunnel, catalyzing it processively without being detached [81]. Furthermore, detached single chains are prevented from reassociating with the insoluble polymer, thereby improving efficiency [78,81]. Aromatic residues (Figures 2 and 3) and lining the roof of chitinases with tunnel-like clefts are thought to enable the substrate to slide down the cleft during successive hydrolytic rounds before they are detached [81].

Processivity is measured by comparing the production of soluble and non-soluble reducing ends or by roughly assessing the ratio between produced dimers and monomers [82]. Studies on the chitinase machinery of the model *S. marcescens* have demonstrated that S*m*ChiA and B processively degrade chitin yielding dimers in an exo-hydrolytic mode [22,56,69] (reviewed in [16]). They might yield odd number oligomers like monomers, trimers, and pentamers by an initial endo-active binding. Rehydrolysis of such odd-numbered oligomers results in more even-numbered oligomers in addition to few odd-numbered oligomers [16,82].

*Sm*ChiC is non-processive, randomly cleaving the substrates, and dissociating after every round of hydrolysis yielding odd- and even-numbered oligomers in equal amounts [74,79]. Non-processive GH 18 enzymes like *Sm*ChiC have shallow clefts with fewer aromatic residues (Figure 4), thus enabling more flexibility within the catalytic site that enables detachment and reattachment in disordered regions of the chitin polymer [83]. Payne et al. [83] observed a more dynamic binding of *Sm*ChiC2 in the catalytic site as expected of non-processive chitinases and suggested that the difference in degree of processivity among *Sm* chitinases has more to do with the product subsite than the substrate subsite that also consists of some of the conserved aromatic residues required for catalysis.

Plant GH 18 chitinases that bear high structural similarities with the processive bacterial chitinases have been found to be non-processive. *Nt*ChiV with an RMSD of 1.72 when superimposed on *Sm*ChiB was reported to degrade hexa-N-acetylglucosamine [(NAG)_6_] non-processively, even though the catalytic residues were well superimposed. The difference in both chitinases was found to be in the shortening of two loops in the core domain and the CID relative to *Sm*ChiB. It was further observed that some aromatic residues Trp97, Phe190, and Phe191 in the substrate-binding groove involved in binding the NAG residue at +1, +2, and +3 subsites in *Sm*ChiB were substituted with Gly, Pro, and Arg respectively in *Nt*ChiV [34].

Processivity of chitinases is considered beneficial for the hydrolysis of crystalline chitin. However, with soluble substrates, it may be a rate-limiting process because a steady attachment of enzyme to substrate to keep products from reassociating with the chitin chain is not required [78]. Therefore, the choice of processsive chitinases in any downstream application (e.g biomass conversion) comes at a cost of enzyme speed that can be avoided if crystalline substrates can be disrupted by other means to enhance substrate accessibility by non-processive chitinases [78,82].

### Hydrolysis versus transglycosylation

Hydrolysis and transglycosylation (TG) are two significant processes that may determine the suitability of chitinases for specific roles. Chitinases interact with chitin either hydrolytically or by TG and some uniquely exhibit both properties [84]. Hydrolysis (Figure 8C) results in the generation of monomeric or dimeric glucosamine units with low degree of polymerization (DP) [24]. TG on the other hand is a kinetically controlled mechanism exhibited by glycosidases. It has been reported mostly among GH family 18 chitinases and there is no evidence of its existence in GH family 19 [85].

TG takes place when retaining glycosidases trigger the transfer of a glycosidic residue (instead of a nearby nucleophilic water molecule in hydrolysis) from a donor to an acceptor yielding longer chain COS with high DP (Figure 8C) [24]. TG occurring during hydrolysis may be undesirable because it interrupts bond cleavage efficiency and causes misevaluation of hydrolytic activity because of the production of unexpected enzymatic products [86]. *Serratia proteamaculans* ChiD (*Sp*ChiD) was found to exhibit ‘transglyco-hydrolytic’ activity with a hyper-TG activity on DP2 to DP6 substrates, thus generating long chain COS of DP up to 13 and a hydrolytic property that breaks down DP8 and DP10 TG products to DP2 and DP4 COS respectively [24]. TG can however be of advantage if the chitinase has a hyper-TG activity like *Enterobacter cloacae* chitinase designated *Ec*Chi1, reported to produce long chain COS up to DP9 [84].

**Figure 9:**
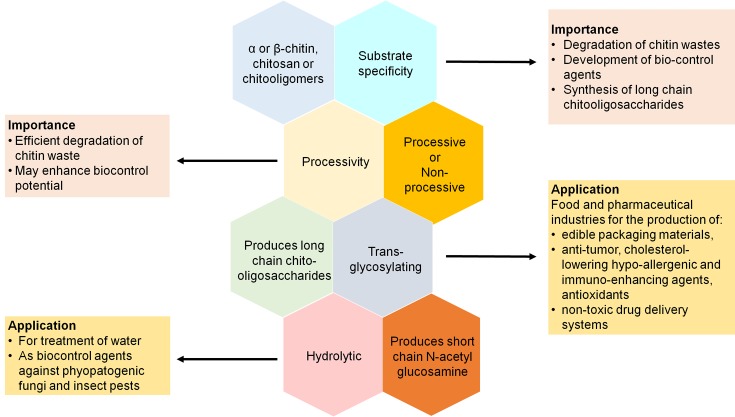
Substrate specificity and accessibility, hydrolysis/TG, and processivity can be modulated for suitable industrial applications

Active site structure and arrangement of catalytic residues [17,87], enzyme concentration [24], nature and concentration of starting substrates [88,89] as well as process duration are factors that have been reported to contribute to the switching ‘on’ or ‘off’ of hydrolytic or TG modes in some chitinases. In addition, chitinases can be engineered to influence activity in favor of hydrolysis or TG depending on the requirement that meets specific commercial applications as represented in Figure 9. Hydrolytic chitinases may be of better application in agriculture as biocontrol agents and in the environment for cleaning up of chitinous wastes [10–12]. TG chitinases, on the other hand, are more relevant for application in food and pharmaceutical industries as food additives or for drug delivery respectively due to their ability to convert crystalline chitin to soluble long chain oligosaccharide [87–89].

## Variation in substrate specificity

Chitinases have varying affinities for different chitinous substrates. Crystalline chitin, the most abundant, is a white, rigid and inelastic polysaccharide available in three different forms. These structures differ in the parallel or antiparallel arrangement of the chitin chain [90,91], which are held together by glycosidic bonds; α-chitin, which is the most abundant form of chitin, has a tightly packed antiparallel arrangement of succeeding sheets. β-Chitin has a less compact, unstable arrangement with parallel chains held together by weaker intermolecular forces and is more readily hydrolyzed than α-chitin [90]. At last, it is the γ-chitin that is polymorphic in nature with a combination of parallel and antiparallel chain arrangement [90,91]. Fungal cell walls, crab limbs, and shrimp shells are composed of α-chitin [45] while both α and β forms are found in insects [2].

Chitinases bind to other less crystalline substrates like chitosan (deacetylated chitin), CC, or synthetic analogues of N-acetyl chitooligosaccharides. These substrates have been extensively used to study the binding properties of chitinases and to describe their substrate preferences. The differences in substrates preferences (crystalline, acetylated, or soluble) among chitinases have been associated with the substrate-binding modules. Chitinases having a CBD and aromatic residues are reported to be more efficient in degrading crystalline chitin, while those lacking a CBD may prefer less crystalline forms [72,73,80]. Also, the length and depth of catalytic clefts and substrate-binding sites in the catalytic domain seem to contribute largely to substrate specificity [61,62,68].

Specific activity of *Paenibacillus barengoltzii* Chi70, a family 18 chitinase with two fibronectin-like domains and a chitin-binding domain, was analyzed against CC, powdered chitin and synthetic COS [5]. High specific activity was recorded against colloidal (30 U mg^−1^) and swollen chitin (28.5 U mg^−1^). It recorded lowest activity toward powdered chitin (0.5 U mg^−1^). The enzyme also exhibited increasing activity with increasing polymeric chains of soluble COS, including (GlcNAc)_3_ (13.5 U mg^−1^), (GlcNAc)_4_ (74.3 U mg^−1^), and (GlcNAc)_5_ (213.4 U mg^−1^). Due to its ability to produce (GlcNAc)_2_ from the hydrolysis of CC, this enzyme was proposed as a good candidate for the industrial production of the oligomer [5].

Suginta et al. [92] compared the kinetic parameters of *Vibrio harveyi* ChiA and its W275G and W397F mutants. Both mutations were reported to have changed the substrate specificity of the wild-type enzyme. W275G lead to a decrease in the *k*_cat_ and *k*_cat_/*K*_m_ toward (NAG)_5_ and (NAG)_6_ by a magnitude of 5, while W397F lead to an increase in the *k_cat_*by magnitudes of 10 and 16 and *k*_cat_*/K*_m_ by magnitudes of 8 and 16 toward (NAG)_5_ and (NAG)_6_ respectively (Table 2). Both mutants however had a reduction in efficiency toward CC indicating the importance of both residues for binding of insoluble substrates. Further evidence on the specificity toward CC was revealed when binding assays were conducted to assess the binding of *Vibrio harveyi* ChiA and its isolated N-terminal Chbd_VhChiA_ (CBD) to both α and β (colloidal and crystalline) chitin and chitosan. ChiA, which has a deep and long binding cleft consisting of six N-acetylglucosamine subsites, showed greater affinity for the colloidal and N-acetylated crystalline, less compact β-chitin over the tightly packed α-(colloidal and crystalline) chitin and chitosan [92].

**Table 2:** Kinetic parameters of some chitinases

Sources and names of chitinases	Substrate	*K*_m_	*k*_cat_	*V*_max_	*k*_cat_ */K*_m_	Reference
*Serratia marcescens*						
ChiA	4-MU-(GlcNAc)_2_	[S]_0.5_ = 135 µM	104 s^−1^	–	–	[69]
	4-MU-(GlcNAc)_3_	4.29 ± 0.4 µM	67 ± 3 s^−1^	*–*	*–*	[68]
ChiB	4-MU-(GlcNAc)_2_	34.1 ± 1.4 µM	19.1 ± 0.7 s^−1^	*–*	*–*	[69]
	4-MU-(GlcNAc)_3_	6.8 ± 1.0 µM	57 ± 4 s^−1^	*–*	*–*	[68]
ChiC	4-MU-(GlcNAc)_3_	80 ± 8 µM	2.0 ± 0.1 s^−1^	*–*	*–*	[68]
Barley chitinase	4-MU-(GlcNAc)_3_	33 µM	0.36 min^−1^	12 µmol min^−1^mg^−1^	1.7 × 10^2^ M^−1^ s^−1^	[125]
	(GlcNAc)_4_	3 µM	35 min^−1^	1.2 µmol min^−1^mg^−1^	1.9 × 10^5^ M^−1^ s^−1^	[125]
*Vibrio harveyi* ChiA	(GlcNAc)_5_	380 ± 49 µM	0.2 s^−1^	–	5.5 × 10^−4^ s^−1^ µM^−1^	[92]
	(GlcNAc)_6_	174 ± 23 µM	0.19 s^−1^	–	11 × 10^−4^ s^−1^ µM^−1^	[92]
	CC	12 ± 1.4 mg ml^−1^	0.10 s^−1^	–	83 × 10^−4^ s^−1^ mg^−1^ ml^−1^	[92]
*Trichoderma atroviride* Ech30	4-MU-[GlcNAc)_2_	149 ± 29 µM	0.0048 ± 0.0004 s^−1^	–		[93]
*Serratia proteamaculans Sp*ChiD	Colloidal	83 mg ml^−1^	3.9 × 10^2^ h^−1^	–-	4.7 h^−1^mg^−1^ ml^−1^	[24]
	(GlcNAc)_2_	203 µM	1.3 × 10^2^ h^−1^	–	0.62 h^−1^ µM^−1^	[24]
*Bacillus licheniformis* ChiA-65	*p*-NP-(GlcNAc)_2_	0.646 mg ml^−1^	24501 s^−1^	36752 U mg^−1^	37927 ± 9.45	[26]
	CC	0.385 mg ml^−1^	5000 s^−1^	7500 U mg^−1^	12987 ± 6.45	[26]
*Bacillus thuringiensis* BthChi74	4-MU-(GlcNAc)_2_	159 ± 0.5 µM	1.5 ± 0.02 s^−1^	–	0.01 s^−1^µM^−1^	[126]
*Bacillus circulans Bc* ChiA1	*p*-NP-(GlcNAc)_2_	2.2 ± 0.5 mM	378 ± 65 min^−1^	–	–	[112]
*Streptomyces coelicolor Sc*ChiC	Crab shell chitin	0.4 mg ml^−1^	–	1207 U mg^−1^	–	[113]
*Enterobacter cloacae* EcCh1	CC	15.2 mg ml^−1^	0.16 × 10^2^ min^−1^		0.011 × 10^2^ mg^−1^ml min^−1^	[84]
	Chitobiase	213.2 μM	1.41 min^−1^		0.6 × 10^−2^ min^−1^ µM^−1^	[84]
*Nepenthes alata* NaCHIT1	(GlcNAc)_4_	1.0 mM	6.8 s^−1^		6.8 s^−1^ mM^−1^	[127]
	[GlcNAc]_5_	2.5 mM	57.2 s^−1^		22.9 s^−1^ mM^−1^	
	[GlcNAc]_6_	2.1 mM	80.7 s^−1^		38.4 s^−1^ mM^−1^	

Abbreviation: *p*-NP, *p*-nitrophenyl.

In addition to the above studies, some comparative studies on *S. marcescens* chitinases have demonstrated differences in the hydrolytic patterns of *Sm*ChiA, *Sm*ChiB, and *Sm*ChiC on soluble and insoluble substrates [22,79]. Among the three enzymes, *Sm*ChiA and *Sm*ChiB were able to accommodate longer chitooligomeric chains [68,69] and *Sm*ChiC was found to have the lowest catalytic efficiency toward short length substrates [68]. Thin-layer chromatography and HPLC analysis of hydrolytic products revealed differences in hydrolytic patterns of *Sm*ChiA, B, and C on chitotetraose [(GlcNAc)_4_] and its synthetic analogue, 4-methylumbelliferyl-β-D-N,N,N′- triacetyIchitotrioside [4MU-(GlcNAc)_3_]_._ While both ChiA and C enzymes cleaved (GlcNAc)_4_ to yield two (GlcNAc)_2_ dimers, the synthetic analogue was cleaved at the 4MU end yielding 4MU plus (GlcNAc)_3._ This was not so with vChiB that cleaved the same substrate in the middle yielding 4MU-(GlcNAc) plus (GlcNAc)_2_ [68]. Among all three chitinases, *Sm*ChiA has the highest activity toward insoluble chitin that may be due to easier accessibility to the crystalline substrate over the other chitinases. Kinetics of *Sm*ChiA and B revealed that both enzymes were inhibited by high substrate concentration. However, at lower substrate concentrations of 4MU-(GlcNAc)_2_, a sigmoid kinetics indicating cooperativity in *Sm*ChiA was observed while a normal hyperbolic kinetics with a *K*_m_ of 34.1 ± 1.4 µM and a *k*_cat_ of 19.1 ± 0.7 s^−1^ was recorded for *Sm*ChiB [69]*. Trichoderma viridae* chitinase designated as Ech30 was observed to exhibit low activity toward short substrates 4MU-(GlcNAc)_2_ (*K*_m_ = 149 ± 29 µM and *k*_cat_ = 0.0048 ± 0.0005 s^−1^). Activity toward β-chitin was higher, yielding high amounts of trimers and tetramers [93]. *Streptomyces coelicolor* ChiG, a family 19 chitinase has no hydrolytic property toward the substrate analogues, 4-MU-(GlcNAc)_2_ or 4-MU-(GlcNAc)_3_. However, it hydrolyzed α and β-chitin, COS and chitosan, although at a slower rate than *Sm*ChiA and B [77]. These observations prove that the nature and length of substrates influence chitinase efficiency in any practical application and a wrong chitinase on a wrong substrate will mean poor efficiency.

## Implications of variations among chitinases for optimization and practical application

### Complexity in optimization

The diverse characteristics of chitinases ranging from the presence and location of multiple domains [21,43], variation in the architecture of their catalytic clefts [80,94], presence of extra loops and aromatic amino acid residues, degree of processivity [81,83], differences in hydrolytic or transglycosylating mechanisms [87], to the nature of products formed [22,42,69] highlight the complex relationships between these characteristics and their roles in chitin degradation. This makes their selection and justification to be used in specific practical applications difficult. For instance, processive *Sm*ChiB might be useful in the hydrolysis of chitin for marine waste management, *Sp*ChiD might be more suitable for the synthesis of long chain COS because of their transglycosylating property [88], and Chi42 from *T. artroviridae* might be more suitable as a biocontrol agent [95]. In other words, there is no ‘one-size-fits-all’ approach to their application for biotechnological purposes. In addition, variations result in complex optimization protocols for chitinase production. Therefore, analyzing and matching catalytic efficiencies between laboratory and field applications may be cumbersome and may yield unstable and inconsistent performances [96]. Variables such as nature of substrates, pH, and temperature as well as inducers and inhibitors with respect to specific chitinases must be well considered for optimizing the efficiencies of chitinases for practical applications. There are evidences that allosamidin, caffeine, and argadin are potent inhibitors of family 18 chitinases [48,97]. However, family 19 chitinases are not known to be affected by any of these inhibitors. More in-depth knowledge on these specific characteristics will ease overcoming these complexities for the development of efficient chitinase systems useful for biotechnological purposes.

### Varied spectrum of biocontrol potential

In agriculture, the inhibitory properties of chitinases are being harnessed for the biological control of pests and fungal diseases of plants. The control of fungal phytopathogens such as *Trichoderma reesei* [98], *Colletotrichum gleosporoides* [99], *Phoma medicaginis* [100], *Rhizoctonia solani* [101], *Fusarium oxysporum* [102], and *Fusarium graminarium* [103] has been reported.

GH family 19 chitinases have been well documented to have appreciable inhibitory properties against fungal phytopathogens than the GH 18 chitinases. This may be expected since they are known to be produced as a defense mechanism against fungal pathogenic attack as demonstrated by Kawase et al. [42] However, some variations in the antagonistic behaviors exist among members possibly due to the differences in their structural and mechanistic features. Class I chitinases with an extra CBD demonstrated ~5-fold higher inhibition than class II chitinases that lack the extra domain. Taira et al. [104] attributed this to the presence and basic nature of the CBD in class I chitinase of rye seeds, which effectively inhibit the fungal pathogen, *T. reesei*, better than the acidic class II chitinase from the same species.

In addition, chitinases have varying spectrums of antifungal properties against different fungi and sometimes do not display any toward other fungal species. This has been demonstrated through growth inhibition assays and chitinase overexpression in transgenic plants exposed to fungal diseases (reviewed in [39,62]). Their inhibitory patterns against filamentous and non-filamentous fungal phytopathogens also differ greatly. Karthik et al. [105] reported that a *Streptomyces* sp. chitinase did not show any effects against *Candida albicans* even though appreciable inhibition was recorded toward all filamentous fungi tested. Barley chitinase that was tested against 15 notorius plant phytopathogen inhibited only six including *Botytis cinerea, Pythium theae, Bipolaris oryzae, Alternaria* sp., *Curvularia lunata*, and *R. solani*. No inhibitory effects against the remaining nine species (*Fusarium solani, Colletotrichum falcatum, C. gleosporoides, Magnaporthe grisea, Sarocladium oryzae*, and *Macrophomina phaseolina, Cylindricoladium scoparium, Cy. xoridanum, Pestalotia theae*) were recorded [63]. Yan et al. [106] reported the varied pattern of antifungal property of a recombinant rice chitinase gene that efficiently inhibited *Rizopus stolonifer* and *Botrytis squamosal* but showed no significant effect on *Aspergillus niger* and *Pythium aphanidermatum*. Also recently, a hyphal inhibition assay was conducted using recombinant *Escherichia coli* BL21 strain harboring a newly isolated 53.3 kDa chitinase (*Pt*Chi19) from *Pseudoalteromonas tunicata* CCUG 44952T, against some pathogens. Among all the pathogens in the phylum Ascomycota tested, growth inhibition was only detected against *F. oxysporum, A. niger*, and *Armillaria mellea*, while no inhibition was observed for *Alternaria solani* and *Botryotinia fuckeliana* [107]. Ech30 from *Trichoderma atroviride* also showed no inhibitory effects toward all three pathogens tested [93].

These variations in antifungal properties of chitinases can be attributed to the diverse composition of chitin in different fungal species [95,108,109]. Another possibility might be the variation in the exposure of glycosidic chains in the cell wall chitin of different fungi [106]. This suggests that a low inhibitory effect is expected toward a low proportion of fungal cell wall chitin or poorly exposed glycosidic bonds. The undefined spectrum of antifungal chitinases can be a downside for their application as biocontrol agents considering the imminent need for new and safer biocontrol alternatives with broad spectrum of activity against diverse plant phytopathogens.

### Implications for the degradation of biomass and synthesis of chitooligosaccharides

Chitinases are also useful for the degradation of chitin from biomass to give forth COS, chitosan, and other chitin derivatives. Marine wastes constitute several billions of tons of chitin deposits in the environment. These wastes can be managed by an efficient enzymatic system of chitin degradation and bioconversion into soluble monomers and COS for use in the food and chemical industries [3,110,111]. COS in the food industry can serve as food additives, product enhancers [3,110], and dietary supplements for building the immune system [112]. In the pharmaceutical industries and in medicine, COS have hypoallergenic, antitumor, antioxidant, and wound healing properties that render them fit for the treatment of tissue-related diseases, development of drug delivery systems, and in the manufacture of surgical materials and implants [3,113].

Several chitinases have been identified as being suitable for COS synthesis. However, their manner of chitin degradation and nature of COS produced in terms of degrees of polymerization, de-N-acetylation and acetylation, is highly varied [110]. For instance, certain chitinases interact with chitin in a hydrolytic manner to yield low molecular weight COS [34,114], while some utilize a transglycosylating mechanism for the addition of oligomeric units to yield longer chain oligomers [24] and others may have a combination of both mechanisms [86]. Therefore, the enzymatic degradation of crystalline chitin to give forth COS is not economically feasible because it might require an industrially complicated process involving the use of a combination of chitinases that are costly and not readily available [110]. This is a major limitation to the application of chitinases for enzymatic degradation of chitin.

### Harnessing chitinases for efficient application by enzyme engineering

Understanding the relationship between the diverse characteristics of chitinases and their functions is necessary for the improvement of functional chitinases that meets specific biotechnological use as represented briefly in Figure 9. Recently, the wide use of molecular methods in the study of microbes and their complex roles in the environment have helped to breakdown some microbial processes controlling chitin degradation [45]. In addition, mutagenesis and enzyme engineering studies as well as the increasing number of solved chitinase structures have been of advantage in providing more insights into specific characteristics. In light of these advancements, few recent studies have been carried out to engineer chitinases for the purpose of elucidating the functions of structural and mechanistic features, improving enzymatic activity or combining features of different chitinases for multiple chitinolytic properties, to ensure or improve their suitability in different applications. Table 3 highlights some attempts and methods that have been applied to engineer chitinases.

**Table 3:** Chitinase engineering studies

Sources and names of chitinases	Mutation technique	Outcome of mutation	Reference
*Beauveria bassinia/Bb*Chit1	Random mutagenesis	Improved catalytic activity and affinity toward CC	[115]
*Bacillus licheniformis* (DSM13 and DSM 8785)/ChiA	Random mutagenesis	Improved catalytic activity	
*Serratia marcescens/Sm*ChiA	SDM	Modulation of processivity	[81]
*Bacillus licheniformis*	Domain fusion	Increased catalytic activity and thermal stability	[121]
*Serratia proteamaculans* ChiD	SDM	Increased catalytic activity and thermal stability and modulation of TG	[88]
*Bacillus pumilis* SG2	Random mutagenesis	Increased affinity to CC	[117]
*Trichoderma atroviridae* Chit42 + CBD of *S. marcescens* ChiB	Domain fusion	Retention of Chit42 catalytic property and increased antifungal activity	[95]
*Serratia proteamaculans* ChiD	SDM	Modulation of TG and hydrolytic properties	[119]
*Trichoderma atroviride* Chit42 catalytic domain + *Trichoderma atroviride* 18-10 CBD	Domain fusion	Increased specific catalytic and antifungal activities	[123]
*Bacillus thuringiensis/* Chi9602	SDM	Increased catalytic and antifungal activities	[118]
*Cycas revoluta* /*Cr*ChiA	SDM	Modulation of TG	[86]
*Pseudomonas aeruginosa* /*Pa*chi	Domain modification	Increased catalytic activity, reduced affinity to CC in the absence of CBD	[124]
*Serratia marcescens/Sm*ChiB	SDM	Changes in thermodynamics for favorable substrate binding and tunnel formation	[57]

### Mutagenesis for improving chitinase activity

Mutagenesis by DNA shuffling and up to three cycles of error prone PCR were performed to generate variants of chitinase *Bb*Chit1 from the fungus *Beauveria bassiana*. Two variants, SHU-1 and SHU-2, were identified with mutations outside of the binding site and catalytic pocket, resulting in an increased catalytic activity toward CC compared to the wild-type chitinase [115].

Similarly, Songsiriritthigul et al. [116] through directed evolution developed a library of mutant chitinase genes from two highly identical *Bacillus licheniformis* (DSM 13 and DSM 8785) ChiA genes by carrying out a combination of error prone PCR and DNA shuffling techniques. A chitinase active variant was identified by a medium-throughput screening of positive *E. coli* transformants harboring the variant genes on medium containing CC. The variant after a second screening was found to have improved catalytic activity *p*-nitropheyl-chitobiose as substrate compared to native ChiA enzyme [116].

A non-PCR based method of mutagenesis involving the use of chemicals and irradiation on microbial cells has also been explored for random mutagenesis of chitinases. A randomly mutated halotolerant strain of *Bacillus pumilis* SG2 (designated as AV2-9) expressing two chitinase genes, ChiS and ChiL, was obtained using UV irradiation and treatment with nitrous acid [117]. Screening of the whole *B. pumilis* SG2 and AV2-9 chitinase operons (promoter and coding sequence spanning from ChiS to ChiL) revealed a codon change from GGA to GAA in ChiL gene sequence leading to an amino acid substitution at position 432 from glycine to glutamic acid. Cloning and expression of the variant gene in *E. coli* further revealed an increased thermal stability and a 30% increase in catalytic activity of the variant over the wild-type ChiL [117].

### Site-directed mutagenesis of chitinases

SDM is a method of choice for mutation studies because of the less number of iterations required and the generation of fewer but more accurate libraries, which eliminate the need for high-throughput screening. Ni et al. [118] introduced mutations by replacing selected amino acid residues in Chi9602 of *Bacillus thuringiensis* with alanine to obtain ten variants harboring a single mutation each. Seven of the mutations were introduced to conserved residues leading to loss of enzyme activity. Only three variants with mutations that involved non-conserved residues. They were ChiW50A that was mutated in the chitin-binding domain; ChiD385A and ChiS450A that were mutated within the catalytic sites. All three variants have higher catalytic activities of 62%, 15%, and 51% respectively compared to the native enzyme, Chi9602. Further analysis of ChiW50A showed increased antifungal activity against *Sclerotinia sclerotiorum, Fulvia fulva, Botrytis cinerea*, and *F. oxysporum*. The study proposed that changes in the depth of the catalytic site of ChiW50A, elimination of hydrophobicity, and increased hydrogen bonding interaction among residues of close proximity in the active site of ChiS450A were possible reasons for the increase in enzyme activity [118].

### Modulation of transglycosylation by SDM

TG is a more efficient and environmental friendly approach involving enzymatic synthesis of oligosaccharides as opposed to a costly and hazardous chemical process that involves chromatographic separation of reaction products at every stage. Some family 18 chitinases have been modified to enhance their TG activities for increased enzymatic production of COS [89]. TG activity was modulated in *Cycas revoluta* chitinase *Cr*ChiA GH by altering the side chain of Trp168 to Ala and Gly77 to Trp respectively. The former inactivated TG activity while the later enhanced TG activity. This study demonstrated the importance of the side chain of aromatic residues to TG activity [86]. TG was improved by altering the substrate-interaction sites involving residues in the catalytic center, catalytic cleft, and solvent exposed region of *S. proteamaculans* ChiD (*Sp*ChiD). Mutated residues lining the cleft lead to increased TG as observed in variants W160A, M226A, Y228A, and N284A that were in close proximity to the catalytic DXDXE motif. Variants F125A, G119S, and S116G with substitutions in the catalytic groove had an extended duration of TG activity that resulted in the formation of products with higher DP [88].

Similarly, Madhuprakash et al. [119] carried out point mutations to determine the importance of selected residues on either side of the catalytic grooves of *Sp*ChiD in TG and hydrolysis of chitin. The results that were correlated with a molecular dynamics simulation revealed the entry and exit points of substrate and products respectively. TG activity of *Sp*ChiD was enhanced when Trp120, which is situated just immediately after the SXGG motif, was mutated to Ala. In contrast, both TG and hydrolytic activities were lost in G119W (last residue in the SXGG motif) mutant due to the hindrance to the movement of the DP4 substrate into the catalytic groove supposedly caused by the bulky tryptophan residue as observed during MD simulation. Also, the mutant G201W exhibited an increased TG activity possibly due to retention of the DP4 substrate or the oxazoline intermediate in the catalytic groove [119]

### Modulation of processivity by SDM

Some reports have demonstrated that processivity can be influenced by point mutations or by deletion or addition of loops to the catalytic site. The catalytic properties of *Sm*ChiA and three variants W167A, W275A, and F396A were studied [44,81]. It was observed that processivity was almost completely lost in the double mutant W167A/W275A toward crystalline chitin but recorded a 20-fold increase toward chitosan. This highlights the importance of aromatic tryptophan residues for chitin binding and degradation. Although processivity has always been related to the active site topology of chitinases, the hallmarks of processivity in GH family 18 was recently analyzed by comparing the recently solved structure of *Sm*ChiC with *Sm*ChiA and *Sm*ChiB. Payne et al. [83] solved the 3D structure of the catalytic domain of *Sm*ChiC (Protein Data Bank, PDB ID: 4AXN) and identified a more dynamic ligand binding, solvation and flexibility in its catalytic system, different from the processive *Sm*ChiA and *Sm*ChiB. Hamre et al. [57] studied the thermodynamics of substrate binding and tunnel formation in processive *Sm*ChiB by comparing the solvation and conformational entropies of mutants with wild-type. The study showed a less favorable change in conformational entropy in four mutants: W97A, F190A, W220A and E221A, an indication that the wild-type residues are important for substrate binding, processivity, rigidity, and shaping of the tunnel upon substrate binding [32].

### Chimeric chitinase design

Chimeric enzymes are designed to mimic natural systems of modular proteins also known as fusion proteins [120]. Domain fusion is now used for purposes including increasing enzyme reaction rates or developing enzymes with dual, multiple, or novel functions [120]. Multidomain chitinases are attractive tools for chitinase engineering studies and the chitin-binding domains of a few have been useful targets for the design of chitinases with multifunctional properties or for improved activity.

Neeraja et al. [121] developed two mutants, BliGH and BliGH–CeBD, by deleting the chitin-binding domain from *B. licheniformis* chitinase and fusing a cellulose-binding domain (CeBD) to the C-terminal of the *B. licheniformis* chitinase respectively using PCR techniques. The hybrid mutant BliGH–CeBD had an increased affinity to CC than the deletion mutant. Using the same approach to fuse CeBD to the C-terminal chitinase of *B. thuringiensis* however yielded different result that was suspected to be due to incompatibility of linkers used [121].

In a similar study, a chimeric chitinase, ChC, was designed by fusing the chitin-binding domain of *Sm*ChiB to the catalytic domain of *Trichoderma artoviridae* chitinase (Chit42) [95]. The chimeric enzyme, ChC, retained the catalytic property of Chit42 toward CC and had higher antifungal property toward plant pathogenic fungi. A 70% increase in affinity and 700 times increase in binding constant toward insoluble chitin were also observed. Studies on the physicochemical properties of the variant enzyme revealed a reduction in the α-helix thus changing the structure of Chit42 which in turn enhanced its thermal and chemical stability [122]. In a separate study, Kowsari and Motallebi [123] constructed a chimeric chitinase by fusing the CBD of *T. atroviride* chitinase 18-10 (AAZ23945.1) to the N-terminal catalytic domain of Chit42 of *T. atroviride.* The fusion was created through Splicing by Overlap Extension PCR. A chimeric chitinase termed Chit42–ChBD with an improved catalytic activity was obtained. The modified chitinase had an overall higher specific catalytic activity up to 390 U/mg compared to the native Chit42 with highest activity of 210 U/mg. Fungal inhibition was also improved from 1.72- to 3.44-fold against *S. sclerotium.*

Chen et al. [124] studied the effect of domain modification on the efficiency of catalysis of *Pseudomonas aeruginosa* chitinase (*Pa*chi) on chitin. Two mutants of *Pa*chi one without the CBD domain (CHA) and the other with CBD and Fn3 domains only (CBD+Fn3-*Pa*chi) were constructed. An increased solubility and expression of CHA without the CBD was observed compared to *Pa*chi and CBD+Fn3-*Pa*chi in which both possessed the CBD. Although catalytic efficiency increased, there was a decrease in affinity toward CC in the absence of CBD [124].

## Conclusion

Although several reports have extensively highlighted the importance of chitinases for various biotechnological and environmental applications, not many have reported successful practical applications. This might be a result of the difficulty in unifying optimization processes owing to the high variability in the characteristics of chitinases. Many attempts have been made as reviewed to enhance the catalytic properties through directed evolution, SDM, and domain fusion. Through these studies more information about the importance of domains, specific loops, and residues in chitinases have been elucidated and can be harnessed for useful chitinase application. Further exploration of domains and their respective functions will be of advantage in designing chitinases with enhanced properties. Studies on the interaction between chitinases and different forms of chitin can also help to better understand the varied antifungal properties of chitinases. The application of molecular dynamics to predetermine and compare the behavior of various chitinases may reveal novel mechanisms of interactions. Consequently, more design and mutation strategies can be employed to develop chitinases with enhanced antifungal, hydrolytic, or transglosylating properties. Finally, the fast-growing field of bioinformatics, the availability of more computational tools, and the increasing number of databases describing and predicting protein structure and function can help to streamline engineering of chitinases in a more rational manner to yield chitinases with improved activity and stability specific for the various biotechnological applications.

## Comepting Interests

The authors declare that they have no competing interests.

## Abbreviations

4MU-: 4-methylumbelliferyl-
CBD/ChBD: chitin-binding domain
CBM: carbohydrate-binding module
CC: colloidal chitin
CeBD: cellulose-binding domain
CID: chitin insertion module
COS: chitooligosaccharides
DP: degree of polymerization
Fn_3_LD: fibronectin III-like domain
GH: glycosyl hydrolase
GlcNAc: N-acetyl-d-glucosamine
PDB: Protein Data Bank
PDBe: Protein Data Bank in Europe
*p*-Np: *p*-nitrophenyl
SDM: site-directed mutagenesis
TG: transglycosylation
TIM: triosephosphate isomerase
